# Characterization of Neutralizing Profiles in HIV-1 Infected Patients from whom the HJ16, HGN194 and HK20 mAbs were Obtained

**DOI:** 10.1371/journal.pone.0025488

**Published:** 2011-10-10

**Authors:** Sunita S. Balla-Jhagjhoorsingh, Betty Willems, Liesbeth Heyndrickx, Leo Heyndrickx, Katleen Vereecken, Wouter Janssens, Michael S. Seaman, Davide Corti, Antonio Lanzavecchia, David Davis, Guido Vanham

**Affiliations:** 1 Institute of Tropical Medicine, Antwerp, Belgium; 2 Division of Viral Pathogenesis, Beth Israel Deaconess Medical Center, Boston, Massachusetts, United States of America; 3 Institute for Research in Biomedicine, Bellinzona, Switzerland; 4 Biomedical Primate Research Centre, Rijswijk, the Netherlands; 5 Department of Biomedical Sciences, University of Antwerp, Belgium; 6 Faculty of Medicine and Pharmaceutical Sciences, Free University of Brussels, Belgium; Agency for Science, Technology and Research - Singapore Immunology Network, Singapore

## Abstract

Several new human monoclonal antibodies (mAbs) with a neutralizing potential across different subtypes have recently been described. Three mAbs, HJ16, HGN194 and HK20, were obtained from patients within the HIV-1 cohort of the Institute of Tropical Medicine (ITM). Our aim was to generate immunization antibodies equivalent to those seen in plasma. Here, we describe the selection and characterization of patient plasma and their mAbs, using a range of neutralization assays, including several peripheral blood mononuclear cell (PBMC) based assays and replicating primary viruses as well as cell line based assays and pseudoviruses (PV). The principal criterion for selection of patient plasma was the activity in an ‘extended incubation phase’ PBMC assay. Neutralizing Abs, derived from their memory B cells, were then selected by ELISA with envelope proteins as solid phase. MAbs were subsequently tested in a high-throughput HOS-PV assay to assess functional neutralization. The present study indicates that the strong profiles in the patients' plasma were not solely due to antibodies represented by the newly isolated mAbs. Although results from the various assays were divergent, they by and large indicate that neutralizing Abs to other epitopes of the HIV-1 envelope are present in the plasma and synergy between Abs may be important. Thus, the spectrum of the obtained mAbs does not cover the range of cross-reactivity seen in plasma in these carefully selected patients irrespective of which neutralization assay is used. Nevertheless, these mAbs are relevant for immunogen discovery because they bind to the recombinant glycoproteins to which the immune response needs to be targeted in vivo. Our observations illustrate the remaining challenges required for successful immunogen design and development.

## Introduction

Despite intense research efforts over nearly three decades, only minimal progress has been made in developing an HIV-1 vaccine. In retrospect, a number of reasons can be proposed for this failure such as the enormous genetic diversity of HIV, the camouflage of the neutralizing epitopes in the envelope spike by glycan shields, the presence of “decoy” immunodominant non-neutralizing antigenic determinants in non-conserved areas on the surface and the low gp120 trimer spike density on the virus membrane [1]. In addition, the most vulnerable regions may only be accessible for a short period. These short-lived structures include the so-called CD4 induced (CD4i) in gp120 and the pre-hairpin epitopes in gp41 that are only exposed following CD4 receptor binding and the subsequent conformational changes. Still, a few antibodies (Abs) are able to successfully interfere with the binding and fusion process, as seen in passive immunization studies in the macaque model. Such mAbs include 2G12 (binds to mannose residues on gp120); b12 and F105 (bind to the CD4 binding site, CD4bs); 17b and ×5 (recognize conformational epitopes in the CD4i region); and 4E10 and 2F5 (bind to epitopes in the membrane proximal extracellular region or MPER of gp41). Last year, however, three new mAbs (HJ16, HGN194 and HK20) were reported from African patients from the ITM HIV-1 cohort. Taken together these mAbs target three different steps in viral entry: binding to CD4bs and thus preventing interaction of HIV-1 with CD4 by HJ16, binding to V3 and blocking the coreceptor binding by HGN194 and finally immobilizing the unfolding of the gp41 by the HK20 mAb [2]. Since HK20 targets HR1 instead of MPER or glycans in this region, it has the conceptual advantage over 4E10 and 2F5 of avoiding potential auto reactivity [2], [3]. Importantly, the HGN194 mAb has recently been found to confer protection in infant rhesus monkeys by the group of Ruprecht [4].

In order to generate these mAbs, patient plasma were selected with a neutralization assay with an extended incubation time, using activated PBMC and a panel of clinically isolated replication competent HIV-1 strains. This assay differs from the classical ‘short’ PBMC neutralization assay by extending the incubation phase of plasma with virus from 1 to 24 hours. The importance of this format was shown in a SHIV challenge trial in rhesus macaques, where recombinant HIV envelope immunizations induced protection [5], [6]. Comparing various neutralization assays, we showed that the PBMC based assay with an extended incubation phase was able to discriminate between protected and non-protected animals after vaccination. Since we are attempting to develop a vaccine effective against a range of subtypes and because the subtype A, subtype C and circulating recombinant form (CRF) 02_AG are responsible for at least 75% of the current new infections worldwide, we identified patients, whose plasma could cross-neutralize mainly viruses from these three subtypes in the extended incubation PBMC assay. From the blood of selected patients, memory B cells were isolated and immortalized using an Epstein Barr Virus (EBV) based procedure [7]. Supernatants of B cell clones were tested in ELISA with recombinant gp41, trimeric gp120 and gp140 proteins from several subtypes as solid phase. Clones with binding activity to any of these antigens were expanded and supernatants were tested using a HOS based PV neutralization assay. This effort ultimately resulted in the selection of the new mAbs HJ16, HGN194 and HK20, which showed considerable breadth of neutralizing activity against a panel of HIV-1 primary isolates spanning both tier 1 and tier 2 viruses of different subtypes [2].

Here, we present the characteristics of the patient's plasma and their respective mAbs in multiple neutralization assay formats. The results clearly demonstrate that patient selection was highly dependent on the neutralization assay. Although the cross-neutralizing properties of the isolated Abs showed considerable variation with the neutralization assay format, all assays indicate that neutralizing Abs to other epitopes of the HIV-1 envelope are present in the plasma and also do not exclude the role that synergy between such Abs could play.

## Materials and Methods

### Ethics statement

The study was approved by the Institutional Review Board of the Institute of Tropical Medicine and the Ethical Committee of the University Hospital of Antwerp. All participants understood and signed an informed consent.

### Patient selection

Eligible patients visiting the ITM clinic in Antwerp had been infected for at least one year, were clinically asymptomatic and over 18 years old. Neither CD4 T cell counts nor viral loads were taken into consideration. Patients were preferentially selected from sub-Saharan regions where the subtypes A, C and/or CRF02_AG are prevalent. Plasma was subsequently screened for its ability to neutralize a panel of four subtype A, four subtype C and six CRF02_AG primary HIV-1 strains, in our extended incubation phase PBMC assay (see below).

### Monoclonal antibodies

HJ16, HK20 and HGN194 Abs were obtained as part of the Collaboration for AIDS Vaccine Discovery program from Dr. D. Corti (Institute for Research in Biomedicine, Bellinzona, Switzerland).

### Cells

Buffy coats from healthy donors from the Red Cross Blood Transfusion Center at the University Hospital of Antwerp were used for isolation of PBMC by LymfoPrep (Axis-Shield, Oslo, Norway) centrifugation and adjusted to 1×10^6^/ml in culture medium, consisting of RPMI 1640, 15% fetal calf serum (FCS), 0.03% L-glutamine and 50 µg/ml gentamycin (Lonza, Verviers, Belgium), 2 µg/ml polybrene (Sigma-Aldrich, Bornem, Belgium). Cells were stimulated with 0.5 µg/ml phytohemagglutinin (PHA, Oxoid, Hampshire, UK) for 2 days and 1 day with 200 U/ml interleukin-2 (IL-2; Gentaur, Brussels, Belgium) in a 7% CO_2_ incubator at 37°C and then used for neutralization assays. The following cell lines were obtained through the NIH AIDS Research and Reference Reagent Program, Division of AIDS, NIAID, NIH: TZMbl from Dr. John C. Kappes, Dr. Xiaoyun Wu and Tranzyme Inc. and Ghost(3)X4/R5 from Drs. Vineet N. KewalRamani and Dan R. Littman. HEK 293T cells were obtained from the American Type Culture Collection, ATCC, Virginia, US.

### Replication competent HIV-1 virus and single-cycle HIV-1 pseudoviruses

For patient selection a panel of 14 primary group M isolates representing subtypes A, C and CRF02_AG were used: four subtype A strains (VI 191, 92UG37, VI 820, VI 1031), four subtype C strains (VI 829, VI 882, VI 1144, VI 1358) and six CRF02_AG strains (VI 1090, VI 2680, CI 20, CA 18, VI 1380, VI 2727). All isolates were classified by phylogenetic analysis of their envelope genes. All virus stocks were prepared and titrated on PHA/IL-2 stimulated PBMC. These strains have been extensively used for at least 10 years at ITM and are considered equivalent to neutralization resistant tier 2 viruses [8], [9]. Corresponding envelope PV constructs were obtained by DNA amplification of the complete *env* starting from PBMC co-cultures or by RT-PCR using plasma and subsequent cloning into an expression vector (pSV7d or pcDNA4/TO) [10]. These included the ITM strains VI 191 (A), VI 829 (C), VI 882 (C), VI 1358 (C), VI 824 (D), VI 1888 (CRF01), VI 1090 (CRF02), CI 20 (CRF02) and CA 18 (CRF02). The *env* expressing plasmids 92RW009 (A), SF162 (B) and 92BR025 (C) were provided by the EU Programme EVA Centre for AIDS Reagents, NIBSC, UK (AVIP Contract Number LSHP-CT-2004-503487). Sequencing of the PV constructs and phylogenetic analyses of the complete gp160 confirmed the identity of the PV and its corresponding virus. The full length *env* sequences of the ITM PV constructs have been deposited with GenBank (accession numbers EU191613 for VI 829, EU191617 for VI 1888, EU191618 for VI 191, HQ912706 for CA 18, HQ912707 for CI 20, HQ912708 for VI 882, HQ912709 for VI 824, HQ912710 for VI 1090 and HQ912711 for VI 1358).

### Neutralization assays

Since several parameters influence the observed neutralizing profile of a plasma or mAb, we included a comprehensive range of different neutralization assays with distinctive characteristics. Apart from the difference in target cell (primary cells vs. cell lines), incubation, absorption and culture phases were also investigated as determinants of neutralization outcome. Formats for the different assays are as shown in Table 1.

**Table 1:** Overview neutralization assays.

Assay	Nomenclature	Virus	Incubation period	Absorption period	Culture period
24/1/14 PBMC	extended incubation	Infectious strains	24 h	1 h, then wash	14 days
1/2/7 PBMC	short incubation	Infectious strains	1 h	2 h, then wash	7 days
1/24/14 PBMC	extended absorption	Infectious strains	1 h	24 h, then wash	14 days
TZMbl_IV	primary virus TZMbl	Infectious strains	1 h	whole culture period	2 days
TZMbl_PV	TZMbl-PV	Pseudo virus	1 h	whole culture period	2 days
GHOST_PV	GHOST-PV	Pseudo virus	1 h	whole culture period	3 days
HOS_PV	HOS-PV	Pseudo virus	1 h	whole culture period	3 days

#### PBMC based assays

All PBMC neutralization assays are described as a/b/c where ‘a’ is the incubation time in hours following mixing of mAb with virus, ‘b’ is the absorption time in hours during which the cells are exposed to the mAb/virus mixture. Cells are then washed and ‘c’ is the culture time in days (all at 37°C and 7% CO_2_). In this study results were obtained in 24/1/14, 1/2/7 and 1/24/14 formats. These are named ‘extended incubation’, ‘short incubation’ and ‘extended absorption’ assays respectively. The extended incubation assay, which was originally used for patient selection has been described previously [6], [11]. Briefly, virus stock is diluted in a five fold series from 1/2 to 1/6250 in culture medium (RPMI-1640 medium supplemented with 15% FCS and 200 U/ml IL-2) to establish the titer (-log10 if the dilution at which 50% infection is achieved). A titer below 1 constitutes poor growth of the virus and the experiment is discarded. Ninety µl of each virus dilution are mixed with 5 µl of plasma or 50 µg mAb. In assays testing neutralization by plasma the mixture is complemented with 5 µl culture medium to give a final 1 in 20 dilution of plasma. When testing mAb, the mixture is complemented with 5 µl flow through (IgG was removed from HIV-1 negative plasma using a Protein G column [GE Healthcare Europe GmbH, Belgium]) to give 50 µg/ml of mAb. After the incubation phase 20 µl of each plasma or mAb/virus mix are first dispensed in quadruplicate into flat bottom 96-well microplates and 75,000 PBMC in 100 µl culture medium are added to each well. Plates are then left in a CO_2_ incubator at 37°C during the absorption phase (b). Afterwards, cells are washed three times by centrifugation at 2000 rpm for 10 minutes, the supernatant is aspirated and 180 µl fresh culture medium are added to the cells. When cultured for 14 days, 125 µl of the medium is aspirated and replaced with 135 µl fresh culture medium. After c days, 200 µl of the supernatant are mixed with 50 µl Nonidet P40 (0.25% in PBS; Fluka, Sigma-Aldrich, Puurs, Belgium) to disrupt virions and this mixture is analyzed for the presence of HIV p24 antigen. As a control, pooled plasma of 100 HIV-1 negative donors are tested in parallel. For the short incubation and extended absorption phase assays, times of incubation are appropriately adjusted: 1/2/7 and 1/24/14 respectively. Neutralization activities are presented as the percentage reduction in infectious titer of a virus isolate following incubation with patient plasma or mAb relative to its titer following incubation with HIV-1 negative control plasma. Virus titers were calculated by the method of Reed and Muench [12]. An 80% reduction in titer was considered significant. By extending the usual one hour absorption phase of the extended and short incubation PBMC assay to 24 hours (1/24/14 format) we aimed to reproduce the conditions of the cell line based assays where the mAbs remain during the entire absorption and culture phase.

#### Pseudovirus based assays

Neutralization capacity of patient plasma and mAbs against PV on TZMbl and the HOS cell related GHOST.CD4-X4/R5 cells was determined as described [13], [14]. Luciferase reporter gene activity was quantified 48–72 h after infection upon cell lysis and addition of firefly luciferase substrate (Perkin-Elmer) as described. Emitted relative light units (RLUs) were quantified on a LB941 Berthold luminometer (Alabama, US). Infection of TZMbl cells was quantified using SteadyLite and infection of GHOST cells was quantified using BriteLite as a substrate (both Perkin-Elmer). In a preliminary experiment 1.10^4^ TZMbl or GHOST cells were seeded in each well of 96-well, flat bottom plates and infected with a range of viral doses in a total volume of 200 µl to establish the dose, which resulted in a signal of 50,000 to 100,000 RLU in the presence of 10 µg/ml diethylaminoethyl-dextran (DEAE-dextran, Sigma, Belgium) to enhance virus infectivity in TZMbl cells, while no DEAE-Dextran was used in GHOST cells. In the actual neutralization experiments, mAbs or plasma were pre-incubated with PV for 1 h at 37°C. The mAb concentration or plasma dilution producing a 50% reduction in luciferase reporter gene production was determined by linear regression analysis in Microsoft Office Excel as described on http://www.hiv.lanl.gov/content/nab-reference-strains/html/Protocol-for-Neutralizing-Antibody-Screening-Assay-for-HIV-1-in-TZM-bl-Cells-November-2010.pdf. For IC50 of mAbs, the 50% inhibitory concentrations were determined via a linear interpolation method using the mean of duplicate or triplicate cultures. The assay readouts for the dilutions above and below the IC50 were joined with a straight line, plotted against the log concentration of mAb. The position where the line crossed the 50% assay readout was taken as the IC50 estimate. Where the IC value was outside the range of concentrations tested, it was recorded as either greater than the highest concentration used, or less than the lowest concentration, as appropriate. An ID50 for plasma and IC50 for Abs were calculated from a dilution series starting from 1∶20 for plasma and starting from 50 or 150 µg for Abs depending on the Ab used.

### Statistical methods

The virus titer was calculated within each individual experiment using the method of Reed and Muench [12]. In the virus dilution series, doses ranged between those infecting all cultures (100%) to those infecting none (0%). Wells giving an OD>0.3, against a background of 0.03–0.05 in the ELISA, were considered to be infected. The infectious virus titer was calculated following virus incubation with mAb/plasma. The reduction in titer was calculated as a percentage of the virus titer following exposure to either IgG or plasma which was pooled from 100 HIV-1 negative donors. Purified IgG from this pool was used as the control for mAbs. Correlations were calculated using the Spearman Rank correlation test using Prism version 5.0. Differences or correlations between sets of data were considered significant if *p*≤0.05 and *r*>0.5.

## Results

### Patient neutralization profiles and clinical background

Over 1400 HIV-1 infected individuals regularly attend the clinic at ITM. Of these, 200 patients were identified whose origin was the sub-Saharan regions of Africa where subtype A, subtype C and/or CRF02_AG isolates are prevalent. Their plasma was evaluated when they were therapy naïve or at least 6 months therapy-free for the ability to neutralize primary HIV-1 strains from the A, C and/or CRF02_AG subtypes in the 24/1/14 extended incubation phase PBMC neutralization assay. About 25% of these patients had cross-neutralizing plasma i.e. plasma which neutralized at least 50% of strains belonging to one subtype plus at least 25% of strains from a second. We next classified the best responding plasma according to the HIV subtype they preferentially neutralized: e.g. when at least three out of four of the A or C strains (or five out of six CRF02 strains) gave greater than 80% neutralization. In Table 2 this neutralization profile is shown for 20 patients whose memory B cells were interrogated.

**Table 2:** Neutralizing profile of selected patient plasma against subtype A, subtype C and CRF02_AG isolates in the 24/1/14 extended incubation PBMC assay.

		Subtype A				Subtype C				Subtype CRF02					
Patient code	Subtype	VI 191	92UG37	VI 820	VI 1031	VI 829	VI 882	VI 1144	VI 1358	VI 1090	VI 2680	CI 20	CA 18	VI 1380	VI 2727
HGL-plasma	A	***87,4***	**E**	***97,2***	***95,9***	E	*84,9*	62,0	73,7	*91,1*	78,1	*89,5*	*94,9*	67,6	*97,0*
HGD-plasma	B	***84,9***	***80,9***	***94,8***	***97,7***	55,3	*96,9*	*99,7*	24,1	*91,1*	*86,2*	66,1	*94,2*	10,9	*99,9*
HQ-plasma	CRF02	***98,5***	***97,7***	***96,6***	***90,0***	66,1	*99,3*	8,8	0,0	10,9	E	*99,8*	*97,8*	*98,2*	*99,4*
***HGN-plasma***	CRF02	***80,5***	***96,1***	***87,4***	**55,3**	*91,1*	63,7	74,3	*93,7*	*91,1*	73,7	*88,3*	*97,2*	*96,1*	E
HVDA-plasma	C	24,1	70,5	*86,8*	66,1	**0,0**	***99,9***	***91,1***	***86,2***	*89,3*	66,1	41,1	*90,5*	69,1	0,0
***HK-plasma***	CRF02	*97,7*	62,0	65,3	*99,8*	***99,0***	**32,4**	***95,4***	***96,0***	*98,8*	*88,3*	*97,7*	59,3	*96,4*	69,8
HMB-plasma	A/C	***98,8***	***99,9***	**52,1**	***98,2***	***99,9***	***99,9***	**E**	***80,0***	54,3	*80,0*	*91,1*	*94,8*	55,3	78,1
***HJ-plasma***	C	***96,0***	***98,2***	**60,2**	***97,2***	***85.9***	***99.4***	***99,0***	***94.8***	*94,8*	0,0	93,1	*97,0*	0,0	*84,5*
HGR-plasma	B	***98,7***	***97,8***	***96,0***	***98,7***	***96,0***	***91,1***	***96,9***	**66,1**	*88,8*	*80,0*	71,2	*94,8*	*97,0*	78,1
HMQ-plasma	A/CRF11	73,7	E	69,1	*94,0*	**E**	***92,2***	***83,4***	***96,9***	***99,6***	***93,8***	***96,8***	***97,8***	**75,5**	***98,6***
HU-plasma	B/CRF03/CRF13	***95,1***	***90,0***	***95,9***	***98,9***	***80,9***	***99,2***	***98,8***	**E**	***96,4***	**59,3**	***94,8***	***92,8***	***81,4***	***96,9***
HP/HM/HGM-plasma	CRF02	***88,3***	***98,5***	**47,5**	***97,9***	***80,0***	***98,2***	***99,0***	**65,3**	***99,8***	***88,8***	***90,2***	***99,4***	***99,6***	***91,1***
HGE-plasma	A/CRF02	***99,4***	***97,7***	***98,4***	***99,8***	***96,0***	***98,1***	**66,1**	***97,9***	***99,7***	***93,2***	***99,1***	***99,0***	***80,0***	***99,0***
HY-plasma	A/CRF02	**66,1**	***99,5***	***99,2***	***99,6***	***80,0***	***98,6***	***99,5***	***98,9***	***94,4***	***99,0***	***84,5***	***88,3***	***98,2***	***93,1***
HMV-plasma	C	***99,4***	***98,2***	***99,5***	***94,1***	***91,1***	***96,9***	***98,8***	***99,5***	***99,8***	***91,1***	***99,6***	***99,3***	***98,9***	***98,2***
HL-plasma	CRF02	*80,0*	66,9	53,2	*80,0*	*84,5*	*98,2*	22,4	53,2	*80,0*	63,7	79,1	*99,2*	*94,2*	73,7
HZ-plasma	A/CRF02	42,5	*94,8*	69,1	*93,5*	*94,4*	55,3	E	E	62,0	E	E	*80,0*	*85,9*	E
HGP-plasma	A/CRF02/CRF13/CRF09	41,1	*87,4*	*94,2*	42,5	55,3	76,6	73,7	*90,9*	*99,2*	*91,9*	55,3	30,8	*91,9*	*91,7*
HR-plasma	CRF02	73,7	*80,0*	71,8	79,6	E	*98,6*	55,3	E	*99,5*	E	*98,2*	*80,0*	E	*96,9*
HMA-plasma	A	0,0	E	E	*99,4*	0,0	*88,3*	66,1	*88,3*	*96,9*	*86,2*	74,3	0,0	E	*99,6*

% Neutralization obtained with 1∶20 plasma dilution, ≥80% reduction in virus titer is highlighted in italics, E: enhancement of infection, subtype specific neutralization in bold: ≥80% neutralization with at least 75% of isolates within a subtype.

According to these criteria, four patients' plasma preferentially neutralized subtype A strains (HGL-, HGD-, HQ- and HGN plasma) and two were more specific for subtype C (HVDA and HK plasma). We did not find any patients preferentially recognizing the CRF02 strains. Three of the tested patients neutralized subtypes A and C more than CRF02 (HMB-, HJ- and HGR plasma), one was more C and CRF02 subtype specific (HMQ plasma) and finally five patients displayed broad cross-neutralizing activity over all three subtypes (HU-, HP/HM/HGM-, HE-, HY- and HMV plasma). The remaining five interrogated patients did not display this subtype specific behavior (HL-, HZ-, HGP-, HR- and HMA plasma). There is no obvious association between the subtype infecting a patient and that neutralized by his or her plasma. The patients, from whom the newly isolated mAb were obtained, are underlined in the first column. Remarkably, plasma from these patients showed a rather subtype specific neutralization profile since plasma from patient 242315 (HJ patient) neutralized mainly A and C strains, plasma from patient 314994 (HGN patient) mainly A strains and plasma from patient 529552 (HK patient) mainly C strains. In Table 3 the clinical histories of these patients are summarized.

**Table 3:** Clinical information ITM patients.

Patient code	Donor subtype	Patient origin	Age	Years after 1^st^ diagnosis	ViremiaRNA copies/ml	CD4 countcells/ul	HAART
242315-HJ	C	Democratic Republic of the Congo	45	12	62	277	2003–2005, 2007-onwards
314994-HGN	CRF02_AG	Republic of Guinea	41	10	125	765	no treatment
529552-HK	CRF02_AG	Republic of Ghana	31	1	150	623	no treatment

Patient 242315 from whom the CD4bs specific HJ16 mAb was obtained was a 45 year old Congolese woman who had been visiting our clinic since 1996. She received treatment intermittently and consequently had a varying CD4 count and viral load. Her neutralization profile had been obtained using plasma samples taken after stopping anti- retroviral therapy for 6 to 11 months but she was back on therapy at time of memory B cell interrogation for 7 months. Patients 314994-HGN and 529552-HK were not receiving antiretroviral treatment during this study. Patient 314994 from whom the V3 crown specific mAb HGN194 was obtained was a 41 year old woman from the Republic of Guinea who has been regularly attending our clinic since 1998. She has always maintained low viral loads and high CD4 T counts so far without treatment. Her viral loads varied between undetectable and 2,700 RNA copies/ml while her CD4 counts have fluctuated between 550 and 960 cells/µl. Patient 529552 whose HK20 mAb is specific for the HR1 region of gp41 was a 31 year old Ghanaian woman. Soon after arrival in Belgium in 2005 she tested positive for HIV. Her viral loads (1.500-40.000 RNA copies/ml) have fluctuated although her CD4 counts (500–800 absolute CD4 T cells/ml) have remained relatively stable. She received HAART therapy for a short time during pregnancy in 2008 (i.e. after sample collection for the present study). Afterwards she resumed relative control of her infection. It is clear that these patients represent different disease profiles.

The neutralization profile of patient plasma was originally determined using a panel of 14 replication competent clinical HIV-1 isolates (four subtype A, four subtype C and six CRF02_AG strains, Table 2). In view of the known neutralization resistance of these isolates [6], [8] they were considered to represent ‘Tier 2 like” strains. In the experiments represented in Table 4, an additional panel of four subtype B, four subtype D and four CRF01_AE strains provided us with an overview of the neutralizing potential of the three selected patient plasma across six subtypes with a total panel of 26 “tier 2 like” strains.

**Table 4:** Neutralization profile of HJ, HGN and HK patient plasma against a panel of 26 primary viruses belonging to 6 subtypes in the 24/1/14 extended incubation PBMC assay.

		24/1/14 PBMC (24 strains)		
Subtype	Strain	242315-HJ	314994-HGN	529552-HK
A	VI 191	**96,0**	**80,5**	**97,7**
	92UG37	**98,2**	**96,1**	62,0
	VI 820	60,2	**87,4**	65,3
	VI 1031	**97,2**	55,3	**99,8**
B	89,6	**91,5**	**91,9**	**96,0**
	93US076	**99,0**	**84,9**	E
	92US077	**91,1**	**80,0**	70,5
	93US143	66,1	**80,0**	0,0
C	VI 829	**85,9**	**91,1**	**99,0**
	VI 882	**99,4**	63,7	32,4
	VI 1144	**99,0**	74,3	**95,4**
	VI 1358	**94,8**	**93,7**	**96,0**
D	VI 656	**84,9**	E	73,7
	VI 693	**84,9**	25,9	E
	VI 824	78,1	66,1	69,1
	VI 865	**89,8**	48,7	**87,1**
CRF01	VI 1249	**88,3**	30,8	38,3
	CA 10	**96,4**	E	**80,0**
	VI 1888	**89,5**	24,1	66,1
	THA92_022	**80,0**	E	32,4
CRF02	VI 1090	**94,8**	**91,1**	**99,8**
	VI 2680	0,0	73,7	**88,3**
	CI 20	**93,1**	**88,3**	**97,7**
	CA 18	**97,0**	**97,2**	59,3
	VI 1380	0,0	**96,1**	**96,4**
	VI 2727	**84,5**	E	69,8

% Neutralization obtained with 1∶20 plasma dilution, ≥80% reduction in virus titer is highlighted, E: enhancement of infection.

As can be observed, the 242315-HJ patient plasma has a very broad neutralization spectrum with 21 of the 26 viruses neutralized, including all the C and CRF01 strains, 75% of the subtype A, B and D strains and 67% of CRF02 strains. The 314994-HGN plasma has a narrower range, neutralizing 13/26 viruses, including all of the B strains, 75% of the A strains, 67% of the CRF02 strains, 50% of C strains, but none of the D nor CRF01 strains. The 529552-HK plasma neutralized 12/26 viruses, including 75% of the C strains, 67% of the CRF02 strains, 50% of the A strains and 25% of the B, D and CRF01 strains.

### Influence of neutralization assays on plasma neutralization profile of the three patients from whom the new antibodies were isolated

In order to illustrate the influence of different assays on the neutralization spectra of these selected plasma, we compared results from all the assays shown in table 1 (except for the HOS-PV assay). The virus panel used in this comparison consisted of nine strains from our primary selection panel from which PV were also available. Three strains from the standardized “NeutNet” panel were added: A (92RW009, tier 2), B (SF162, tier 1A) and C (92Br025, tier 2) [15] and personal communication). Results are shown in Table 5.

**Table 5:** Neutralization profile of patient plasma in different HIV-1 neutralization assays.

		Infectious virus				Pseudo virus	
242315-HJ		24/1/14	1/2/7	1/24/14	TZMbl_IV	TZMbl_PV	GHOST_PV
A	VI 191	**96,0**	E	E	8,8	19,5	8,0
	92RW009	E	**80,0**	32,4	16,2	33,8	16,7
B	SF162	**99,8**	**97,4**	73,7	**87,2**	**93,8**	**81,5**
C	VI 829	**85,9**	0,0	24,1	46,6	**79,6**	**82,0**
	VI 882	**99,4**	E	E	**59,7**	**81,2**	**83,4**
	VI 1358	**94,8**	30,8	71,8	30,1	49,2	**69,7**
	92Br025	**97,7**	**80,0**	**94,2**	43,9	**75,4**	**59,9**
D	VI 824	78,1	E	0,0	17,1	18,4	46,5
CRF01	VI 1888	**89,5**	**86,8**	E	26,6	16,9	38,6
CRF02	VI 1090	**94,8**	**88,5**	0,0	41,7	48,1	43,0
	CI 20	**93,1**	71,8	0,0	**54,7**	**66,7**	**58,6**
	CA 18	**97,0**	**96,0**	66,1	**77,6**	**91,2**	**82,4**

		Infectious virus				Pseudo virus	
314994-HGN		24/1/14	1/2/7	1/24/14	TZMbl_IV	TZMbl_PV	GHOST_PV
A	VI 191	**80,5**	E	E	E	25,8	46,0
	92RW009	E	66,1	32,4	16,5	28,6	**63,9**
B	SF162	**99,8**	70,5	**96,9**	**88,5**	**100,3**	**95,2**
C	VI 829	**91,1**	E	E	E	33,2	**59,5**
	VI 882	63,7	E	E	E	E	42,9
	VI 1358	**93,7**	E	68,4	E	E	18,6
	92Br025	**97,7**	55,3	**84,5**	49,3	**83,8**	**86,5**
D	VI 824	66,1	E	0,0	E	E	36,5
CRF01	VI 1888	24,1	49,9	60,2	13,9	E	**52,0**
CRF02	VI 1090	**91,1**	0,0	**84,9**	46,7	**94,7**	**92,4**
	CI 20	**88,3**	E	22,4	26,7	44,4	**74,3**
	CA 18	**97,2**	47,5	62,8	**60,4**	**82,3**	**83,3**

		Infectious virus				Pseudo virus	
529552-HK		24/1/14	1/2/7	1/24/14	TZMbl_IV	TZMbl_PV	GHOST_PV
A	VI 191	**97,7**	0,0	E	E	**79,3**	**94,0**
	92RW009	41,1	E	E	**63,2**	11,6	**57,5**
B	SF162	**99,9**	**91,5**	**90,9**	**93,6**	**100,0**	**97,5**
C	VI 829	**99,0**	55,3	41,1	E	**98,0**	**97,1**
	VI 882	32,4	54,3	0,0	E	**94,7**	**96,9**
	VI 1358	**96,0**	**80,0**	**85,9**	E	**94,3**	**95,8**
	92Br025	**99,5**	55,3	**93,2**	**93,3**	**98,2**	**86,9**
D	VI 824	69,1	45,0	E	E	**52,2**	**95,3**
CRF01	VI 1888	66,1	70,5	30,8	E	**67,0**	**90,4**
CRF02	VI 1090	**99,8**	55,3	0,0	**80,1**	**50,5**	**85,2**
	CI 20	**97,7**	55,3	22,4	E	**97,2**	**96,8**
	CA 18	59,3	**93,8**	E	33,0	**80,7**	**57,7**

% Neutralization obtained with 1∶20 plasma dilution, ≥80% reduction in virus titer is highlighted in bold for the PBMC assays, ≥50% reduction in virus titer is highlighted in bold for the cell line based assays, E: enhancement of infection.

Comparing the neutralization breadth of the three patient plasma in three variants of the PBMC assay, indicates that the HJ and HK plasma neutralize much fewer viruses and the HGN plasma even loses all significant neutralization capacity in the classical short assay (1/2/7), implying that none of them would have been selected using results from this assay. Prolonging the absorption phase to 24 hours (1/24/14), to more closely resemble the cell line based assays (see table 1), only “rescues” some neutralization with the HGN plasma. No correlation was found between the results obtained in the different PBMC assays using the Spearman Rank correlation test. There was a correlation between results from the 24/1/14 extended incubation PBMC assay and those with the TZMbl assay using replication competent “primary” viruses for the 242315-HJ plasma (*r* = 0.62, *p* = 0.03) but not for the other 2 plasma samples. A stronger correlation (*r*>0.60 for all three plasma) was found for the 242315 and 314994 plasma between the 24/1/14 PBMC assay and the TZMbl_PV assay. The correlation was statistically significant (*p*<0.04). The strongest correlation (*r*>0.69) was observed between the two PV assays (TZMbl-PV and GHOST_PV). Correlations for all three plasma were significant (*p*<0.01).

**Evaluation of plasma vs. antibodies in the 24/1/14 extended incubation PBMC assay.** The neutralization profiles of the plasma are compared with those for their respective mAbs for the 24/1/14 extended incubation PBMC assay in Table 6.

**Table 6:** Evaluation plasma vs. mAbs in the 24/1/14 extended incubation PBMC assay.

plasma vs mAbs		242315-HJ		314994-HGN		529552-HK	
Subtype	Strain	plasma	HJ16 mAb	plasma	HGN194 mAb	plasma	HK20 mAb
A	VI 191	**96,0**	66,1	**80,5**	**80,0**	**97,7**	74,3
	92RW009	E	**96,0**	E	71,8	41,1	**88,3**
	CA 1	**99,6**	E	**94,4**	0,0	**99,6**	22,4
B	SF162	**99,8**	0,0	**99,8**	**84,5**	**99,9**	**88,3**
	MN	E	E	42,5	55,3	55,3	E
	BaL	**98,1**	66,1	**90,5**	79,1	**93,1**	73,7
	89.6	**91,5**	55,3	**91,9**	**88,5**	**96,0**	55,3
C	VI 829	**85,9**	**91,1**	**91,1**	74,3	**99,0**	74,3
	VI 882	**99,4**	E	63,7	22,4	32,4	0,0
	VI 1358	**94,8**	41,1	**93,7**	E	**96,0**	0,0
	92Br025	**97,7**	77,6	**97,7**	**84,5**	**99,5**	**91,1**
D	VI 824	78,1	55,3	66,1	0,0	69,1	55,3
	CI 13	**99,8**	**80,0**	49,9	55,3	**96,9**	42,5
CRF01	VI 1888	**89,5**	22,4	24,1	12,9	66,1	22,4
CRF02	VI 1090	**94,8**	**99,4**	**91,1**	E	**99,8**	0,0
	CI 20	**93,1**	71,2	**88,3**	E	**97,7**	51,0
	CA 18	**97,0**	**80,0**	**97,2**	0,0	59,3	E

Shown is % neutralization with ≥80% reduction in virus titer in bold. Plasma were tested at a 1 in 20 dilution, Abs were tested at a concentration of 50 µg/ml, E: enhancement of infection.

The mAbs clearly neutralized a much more restricted range of isolates than the plasma. 242315-HJ plasma and HJ16 mAb both neutralized the subtype C isolate VI829, the subtype D CI 13 and two of the three CRF02_AG isolates, VI 1090 and CA18. 314994-HGN plasma and HGN194 mAb as well as 529552-HK plasma and HK20 mAb neutralized SF162 (B) and 92Br025 (C) while HGN194 mAb also neutralized VI 191 (A) and 89.6 (B). In addition, a number of qualitative discrepancies were observed in that growth of some viruses was strongly inhibited by the mAb, but enhanced by the plasma (e.g. subtype A 92RW009 with 242315-HJ plasma versus HJ16 mAb) or vice-versa (e.g. CRF02 strains with 314994-HGN plasma versus HGN194 mAb). This type of inconsistency was also observed by the group of Nussenzweig when they compared their new mAbs with results from the original plasma [16]. Some neutralization-sensitive isolates, CA1 (A), MN (B), BaL (B) and CI 13 (D), were added to the panel in table 6. However, there was only a limited increase in range with HJ16 mAb reaching 80% neutralization against CI 13 (D) and HGN194 mAb almost neutralizing BaL (B) to 80%. HK20 mAb had no activity against any of the extra isolates.

### Influence of assays on neutralization profile of the three new antibodies

While the plasma demonstrated their broadest range of neutralization in the 24/1/14 assays they also showed activity in the other PBMC and cell-line assays. Since it is possible that the mAbs could share these activities we extended their evaluation to assays with the different formats (Table 7).

**Table 7:** Neutralization profile of mAbs in different HIV-1 neutralization assays.

		Infectious virus				Pseudo virus	
HJ16		24/1/14	1/2/7	1/24/14	TZMbl_IV	TZMbl_PV	GHOST_PV
A	VI 191	66,1	33,9	E	3,1	E	E
	92RW009	**96,0**	41,1	70,5	**71,2**	**85,1**	**96,5**
B	SF162	0,0	**80,0**	E	E	35,2	8,9
C	VI 829	**91,1**	E	E	13,3	**61,3**	**66,1**
	VI 882	E	33,9	66,1	12,8	19,6	8,9
	VI 1358	41,1	E	E	9,1	11,4	E
	92Br025	77,6	35,4	14,9	13,4	E	E
D	VI 824	55,3	41,1	18,7	3,4	29,4	12,1
CRF01	VI 1888	22,4	**85,2**	74,3	10,6	3,4	7,1
CRF02	VI 1090	**99,4**	**99,4**	**98,5**	**97,2**	**100,0**	**100,0**
	CI 20	71,2	E	E	11,5	22,0	E
	CA 18	**80,0**	E	66,1	41,1	24,3	**67,6**

		Infectious virus				Pseudo virus	
HGN194		24/1/14	1/2/7	1/24/14	TZMbl_IV	TZMbl_PV	GHOST_PV
A	VI 191	**80,0**	70,5	E	E	6,3	35,0
	92RW009	71,8	**84,5**	E	E	E	42,9
B	SF162	**84,5**	**98,6**	**99,5**	33,0	**99,9**	**98,5**
C	VI 829	74,3	E	E	E	**66,1**	**74,6**
	VI 882	22,4	E	42,5	E	E	22,2
	VI 1358	E	4,5	12,9	E	E	24,6
	92Br025	**84,5**	24,1	41,1	5,1	**56,7**	**64,7**
D	VI 824	0,0	49,9	55,3	E	E	26,6
CRF01	VI 1888	12,9	**85,2**	**84,9**	E	E	25,1
CRF02	VI 1090	E	0,0	71,2	E	E	**64,2**
	CI 20	E	24,1	E	E	**61,6**	**52,1**
	CA 18	0,0	E	24,1	E	E	**50,9**

		Infectious virus				Pseudo virus	
HK20		24/1/14	1/2/7	1/24/14	TZMbl_IV	TZMbl_PV	GHOST_PV
A	VI 191	74,3	36,9	E	7,5	12,2	26,0
	92RW009	**88,3**	E	32,4	E	E	21,9
B	SF162	**88,3**	E	75,5	16,7	**82,0**	29,4
C	VI 829	74,3	E	E	17,9	20,7	22,5
	VI 882	0,0	E	66,1	16,2	4,5	**50,6**
	VI 1358	0,0	73,1	E	E	18,1	13,8
	92Br025	**91,1**	35,4	E	22,5	15,9	37,9
D	VI 824	55,3	E	41,1	4,6	31,8	29,5
CRF01	VI 1888	22,4	**91,3**	69,8	23,6	9,4	44,9
CRF02	VI 1090	0,0	41,1	**80,0**	11,8	E	20,3
	CI 20	51,0	E	E	31,0	16,5	17,3
	CA 18	E	E	55,3	21,8	E	31,4

% Neutralization obtained with 1∶20 plasma dilution, ≥80% reduction in virus titer is highlighted in bold for the PBMC assays, ≥50% reduction in virus titer is highlighted in bold for the cell line based assays, E: enhancement of infection.

The range of HIV-1 isolates neutralized by both the plasma and mAbs is greatest in the extended incubation 24/1/14 PBMC assay while only three of the 36 mAb/isolate combinations show significant neutralizing activity in the extended absorption 1/24/14 PBMC assay. The HJ16 mAb neutralizes three (SF162, VI 1888 and VI 1090) of the six isolates (92RW009, 93Br025 and CA18) neutralized by the plasma in 1/2/7 PBMC assays (table 5). The HGN194 mAb neutralizes SF162, VI 1888 and 92RW009 while the corresponding plasma do not produce significant neutralization against any isolate in these assays. The HK20 mAb only neutralizes VI 1888. When the absorption and culture phases of the assay are extended to the 1/24/14 setup, HJ16 still neutralizes VI 1090, HGN194 neutralizes SF162 and VI 1888 while HK20 neutralizes VI 1090 for 80%.

With regard to cell line based assays, there is a good concordance for HGN194 mAb and plasma in the GHOST-PV assay. Four of these HGN194-PV combinations are also neutralizing when the target cells are TZMbl. However, when infectious virus is used 10/12 combinations are enhancing. HJ16 mAb neutralizes 92RW009 and VI 1090 in all three cell-line assays while the corresponding plasma failed to do so in these assays (see Table 5). Remarkably, however, 92RW009 was neutralized by 242315-HJ plasma in the 1/2/7 PBMC assay selectively and VI 1090 was neutralized by HJ plasma in both the 24/1/14 and the 1/2/7 PBMC assay. The HK20 mAb only neutralizes VI 882 in the GHOST_PV assay, SF162 in the TZMbl_PV assay and does not neutralize any infectious virus. There was no consistent statistically significant correlation between the levels of neutralization reached in the 24/1/14 PBMC assay and the others except where only a few isolates were actually neutralized or neutralization levels were low.

### Independent evaluation of plasma and monoclonal antibodies in TZMbl assay

In order to link the present and previous studies, plasma were tested in TZMbl assays against a sub-panel of PV included in supplementary table 2 of reference 7.

Comparisons are presented in Table 8.

**Table 8:** Evaluation plasma vs. Ab in the TZMbl pseudovirus assay.

			242315-plasma	HJ16	314994-plasma	HGN194	529552-plasma	HK20
Tier 1	MW965.26	C	**8552**	>50	**20050**	**<0.02**	**22466**	**17.3**
	SF162.LS	B	**2116**	>50	**1210**	**<0.02**	**3529**	>150
	BaL.26	B	**112**	>50	**109**	**0.10**	**180**	>150
Tier 2	Q168.a2	AD	71	**0,04**	**347**	>50	27	>150
	Q769.d22	A	<20	>50	**721**	>50	<20	**31.5**
	Q461.e2	A	**194**	**0,2**	58	>50	<20	>150
	RHPA4259.7	B	**831**	**<0.02**	<20	**35.4**	<20	>150
	AC10.0.29	B	57	>50	**535**	>50	26	>150
	Du151.2	C	**173**	>50	61	>50	31	>150
	ZM109F.PB4	C	24	**2.9**	43	**2.2**	<20	>150
	96ZM651.2	C	**132**	**0.20**	88	>50	42	>50
	T257-31	CRF02_AG	<20	**17**	64	>50	<20	>150
	CH110.2	CRF07_BC	**204**	>50	<20	>50	<20	>150
	CH181.12	CRF07_BC	69	**43.9**	**107**	**49.6**	40	>150

Plasma were tested at the Beth Israel Deaconess Medical Center with different dilutions starting from 1 in 20, results are shown in ID50 values. Abs were tested at different concentrations starting from 150 µg for their capacity to neutralize HIV-1 PV, results are shown in IC50 values (µg/ml). In bold: plasma ID50 ≥1 in 100, Ab IC50 ≤50 µg (see Mat & Meth).

Again, there were anomalies with mAbs neutralizing isolates which were not neutralized by the corresponding plasma and vice versa. Similarly, the mAbs showed a reduced range of neutralization relative to their corresponding plasma. Plasma from the 242315-HJ patient is very effective against the three tier 1 strains and also against five out of 11 tier 2 strains. In contrast, the corresponding HJ16 mAb is not able to neutralize the tier 1 strains and although effective against six out of 11 tier 2 strains, these are not always the same isolates as neutralized by the plasma. Plasma from the 314994-HGN patient is able to neutralize all tier 1 strains as well as four tier 2 strains while the HGN194 mAb is also able to neutralize the tier 1 strains and three out of 11 tier 2 strains. However, again, these are not always the same strains that are neutralized by the plasma. Plasma from the HK patient is able to potently neutralize the three tier 1 strains but none of the tier 2 isolates while the HK20 mAb is effective against only one of the three tier 1 strains and one of the 11 tier 2 isolates.

The proportion of isolates neutralized by an individual plasma was also markedly dependent on the panel of HIV isolates used. The patients' plasma were initially selected in 24/1/14 PBMC assays and the ITM panel of 14 primary infectious HIV-1 isolates (Table 4). In the smaller, modified panel of PV used in Antwerp (Table 5), the 242315-HJ plasma neutralized six isolates (50%), the 314994-HGN plasma neutralized four (33.3%) while the 529552-HK plasma neutralized all but one (92%) of the PV in the TZMbl assay. The same plasma neutralized eight (57.1%; HJ16), seven (50%; HGN 194) and only three (21.4%; HK 20) of the 14 isolates tested at the Beth Israel Deaconess Medical Center (BIDMC).

## Discussion

The present study is based on an extensive program to employ naturally occurring broadly neutralizing Abs from HIV-infected patients as templates for immunogen design against A, C and CRF02 primary viruses. From the memory B cell interrogation of such patients many mAbs were generated, but only three of these (HJ16, HGN194 and HK20) showed interesting novel broad neutralizing capacity. Since the plasma and ensuing mAb were selected in different neutralization assays, we wanted to explore and understand the behaviour of these exceptional plasma and mAbs in various neutralization assays, based on PBMC or cell lines, using primary infectious viruses or non-replicating PV.

A first observation was that the three patients, from whom the neutralizing mAbs were generated, showed an intermediate breadth of neutralization, preferentially neutralizing subtype A (HGN patient) or C (HJ patient) or A and C (HK patient), which did not correspond with the subtypes of their infection. Another remarkable observation is that they all showed a low viral load without treatment at the time of sampling. Only patient HGN had the profile of a viraemic controller, whereas HJ was a chronic progressor and patient HK was probably still in an early phase.

In the last two years several groups have reported the discovery of new and promising Abs [17], [18], [19]. The HJ16, HGN194 and HK20 Abs obtained by our consortium were amongst those obtained by means of the interrogation of rather chronically HIV-1 infected patients. In the present study, the HGN194 patient was infected for at least 10 years and seemed to naturally control her HIV-1 infection, the HK20 patient may not have been infected for longer than a year and the HJ16 patient regularly required antiretroviral therapy to control her viral load. These data confirm that neutralizing Ab development does not protect against disease progression. Similarly, some of the broadest neutralizing plasma were obtained from patients who urgently required HAART (e.g. HY-plasma table 2, clinical history not shown).

A side-by-side comparison of the different neutralization assays used for characterization of these patients' plasma and the newly isolated Abs showed that the broadest spectrum of strains and subtypes was neutralized in the PV assays as well as in the 24/1/14 extended incubation PBMC assay with primary virus. In contrast, the classical short incubation phase assays as well as the extended absorption phase PBMC assays showed a reduction in the number of neutralized strains. The TZMbl assay using primary virus also showed this restricted profile despite the fact that it has an extended absorption phase in common with the cell line PV assays. Results for the three patient plasma that were selected for their cross-neutralizing capacity in the 24/1/14 PBMC assay correlated with those obtained in the TZMbl_PV assay for only two patients. It is unusual that these two substantially different techniques result in comparable neutralization profiles (own results and [20]). Both PV based assays correlated strongly with each other.

Another observation was that all three isolated Abs have a narrower and partially different neutralization spectrum relative to the corresponding plasma in the extended incubation PBMC and TZMbl PV assays. Results with the HJ16 mAb from the PBMC, TZMbl and GHOST assays show good correspondence while for the HGN194 mAb the GHOST neutralization responses are broader. The HK20 mAb shows little to no neutralization in either the TZMbl or GHOST assays. Neutralization breadth across subtypes is unlikely to be due to endotoxin since plasma are negative in conventional assays where absorption phases (and therefore contact between plasma and cells) are longer [21].

Several factors may be responsible for the reduction in the range of isolates neutralized by the mAbs. One reason could be the polyclonal character of the Abs in the plasma. Cross-neutralization may require interaction between Abs acting at several epitopes. In this scenario, reproducing the range of isolates neutralized by plasma would not be possible when an average of only one to two neutralizing mAb were isolated. This would be buttressed by methods to directly determine the number of individual neutralizing antibody clones in the patient's repertoire. We will also address this issue in new studies but this has also been examined by the group of Guan and Lewis [22]. Obviously, combining more isolated mAb might correspond better to the plasma results when additive and synergistic effects between Abs could be unveiled. Unfortunately, we did not obtain more mAbs for the 242315-HJ and 529552-HK patients. Although we did obtain more mAbs from the 314994-HGN patient, none of these, except for HGN194, were neutralizing in either the HOS or TZMbl assays. Nevertheless, there may be ‘missing Abs’ as has been previously suggested by the groups of Guan and Nussenzweig [16], [22]. However, the ‘non-neutralizing’ mAbs may still be relevant in the wider context since they could have other effector mechanisms such as Ab-dependent cell-mediated virus inhibition (ADCVI) or Ab-dependent cellular cytotoxicity (ADCC) through their FcγRs [23], [24], [25].

An alternative explanation for why relatively few neutralizing mAb were obtained is that the primary screen was binding to monomeric or trimeric envelope protein in ELISA and this procedure might not be optimal. In the Walker study [17], it was shown that the most potent Abs did not bind in an ELISA and even Abs that did bind had a low neutralizing profile. The inference was that specific quaternary protein structures should be used in a primary screening. This issue is being addressed with the most recent samples under interrogation at IRB within our consortium. It should also be noted that the Abs in the plasma probably originate in the plasma cells of the bone marrow while the mAbs are isolated from memory B cells in the circulation. These two cell populations may not produce the same range of Abs. It should be possible to culture individual plasma cells, clone their heavy and light chain variable regions and identify the IgG or IgA repertoires produced.

Selection of the patients from whom the mAbs were isolated was extremely assay dependent. The patients who gave the three interesting mAbs would not have been selected if any of the alternative assays had been used. The influence of target cells on neutralization has already been observed both by us and others [10], [20], [26], [27]. In particular, there is a three way interaction effect between the virus, antibody and target cells. Especially MPER specific Abs are more potent in PBMC based assays [4], [26], [27]. Since our data show that the HJ, HK and HGN patients are more potent in PBMC neutralization assays with an extended incubation phase it could be envisioned that these special patients could have a high proportion of gp41 specific Abs in their plasma.

In the past, naturally occurring cross subtype neutralizing Abs have already been used as templates for immunogen design but in most of these cases patients were selected using either the classical short PBMC assay (1/2/7 format) or PV assays and our results clearly show that a different group of patients is selected by the extended incubation PBMC assay (24/1/14 format). Testing the patient plasma against primary strains is also more stringent since the molecularly cloned PV seem to be more easily neutralized. Hence, we believe that our selection procedure against the primary ITM panel provided us with patients that had more potent responses.

A possible reason for any increased sensitivity of primary vs. pseudo viruses for identifying patients with potent neutralizing Abs could be the higher number of envelope glycoprotein spikes on the primary viruses relative to the PV [20], [28]. An alternative factor might be the density of (co)receptors on target cells, which has been implied by Corti et al who reported potent neutralization by HK20 in the HOS assays but almost no potency in TZMbl assays [2], [3]. Since HK20 recognizes an epitope in the gp41 region this could partially be explained by the high level of CCR5 expression on the TZMbl cells making it more difficult for anti-gp41 Abs to be effective [2], [29]. Also, the pathway employed by PV to enter TZMbl cells may be relevant so that HK20 could have been hindered by events following uptake into an endosome [30].

Since the non-replicating PV constructs could not be assessed in the primary target cells the recent development of molecularly cloned constructs in a Renilla replication competent backbone is certainly a step forward in the development of a standardized PBMC based neutralization assay to assess neutralization in primary cells [31], [32]. It remains elusive whether the HJ16, HGN194 and HK20 mAbs would have been obtained from other patients. HK20 like Abs have been detected through ELISA and although the neutralizing capacity of this fraction was not shown it still provides proof that a significant number of HIV-1 infected patients have responded to the gp41-HR1 region which is only briefly exposed [2], [3].

Even after almost 30 years of HIV research and the ongoing search for correlates of protection, there is still a critical need to determine how effective different types of antibody effector mechanisms can be in prevention of HIV-1 infection. Although many groups have tried to identify which neutralization assay can predict in vivo protection, this issue is still open to debate [33]. In several SIV and SHIV macaque studies neutralizing mAbs have correlated with protection [34], [35], [36], [37], [38], [39], [40], [41], [42], [43], but there are also multiple counter examples [44], [45]. In this context, the most compelling demonstration that pre-existing Abs can be protective comes from passive immunization studies with either IgG or mAbs [28], [34], [38], [39], [40], [41], [42], [43], [46], [47], [48], [49]. The most recent study uses the HGN194 mAb against a SHIV strain containing an ‘early’ envelope and emphasizes the importance of potent neutralizing Abs that confer protection against a heterologous mucosal challenge [4]. The latter is highly significant since future vaccines will need to be effective against these relatively resistant early founder strains before infection is established in vivo [50].

Taken together our observations show that a single neutralizing mAb from each of the three patients does not reflect the major neutralizing spectrum of the patients' plasma and there is no apparent correlation of the mAbs targeting HIV strains belonging to the subtype of virus infecting the patient. It is quite evident that different neutralization assays yield different results and it is still unclear which one is most predictive or suited to obtain neutralizing mAbs. Nevertheless, the strategy used for selection of plasma (in an extended incubation PBMC assay) and selection of mAb (based on ELISA binding and neutralizing capacity in a HOS_PV assay) yielded interesting new mAbs. A better understanding of in vitro neutralization characterizations of patient plasma and Abs and will hopefully lead to more effective ways of discovering new Abs that ultimately can be used for HIV-1 immunogen design and subsequent vaccine development.
